# Intensified Multistep Extraction of Phenolic Compounds from Yerba Mate (*Ilex paraguariensis*) Leaves: A Techno-Economic and Environmental Approach

**DOI:** 10.1021/acsomega.5c10400

**Published:** 2026-02-04

**Authors:** Matheus Samponi Tucunduva Arantes, Sara Mariele Nohr de Lima, Giovana Gonçalves Dusi, Cristiane Vieira Helm, Washington Luiz Esteves Magalhães, Vítor Renan da Silva

**Affiliations:** † Chemical Engineering Department, 28122 Federal University of Paraná (UFPR), Curitiba 80060-000, Brazil; ‡ Embrapa Florestas, Colombo 83411-000, Brazil

## Abstract

In the current work, an alternative intensified methodology for the extraction of phenolic compounds from yerba mate is proposed, considering the use of sequential multistages. Conventional single-stage extraction was optimized in its key parameters (temperature and solid/liquid ratio), and its kinetics were evaluated. The multistage extraction process was evaluated considering two and four stages, considering the results on the antioxidant potential of the final extract. A scale-up simulation was conducted, considering the processing of 25 kg of yerba mate a day, and economic and environmental aspects were considered. The best extraction condition was achieved at 70 °C with 0.5 g yerba mate 100 mL^–1^ water. Intensified multistage processes and conventional ones resulted in final extracts with similar antioxidant potential (493.0–526.5 mg GAE L^–1^), highlighting that the three processes can be used for the obtention of the same product. Regarding the economic aspects, using the multistage extraction processes results in a considerable decrease in the Total Capital Investment of a yerba mate plant extract. Finally, environmental analysis shows that the multistep process is more eco-friendly than the conventional one, as it exhibits considerably lower energy consumption and lower CO_2_ emissions. The intensified multistage extraction process is a green and eco-friendly alternative to conventional extraction processes, with potential for implementation in several plant extract industries.

## Graphical abstract

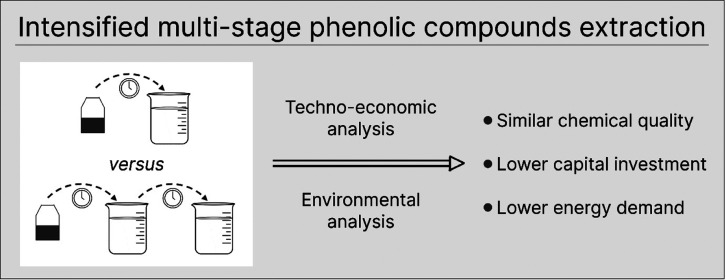

## Introduction

The interest in plant extracts has considerably grown in recent years. Several industries have emerged around the world, for such extracts are highly versatile, and they can be employed in the Food, Cosmetics, and Pharmaceutical segments (e.g., animal diets, food packaging, milk beverages).^1–3^ Phenolic compounds (PC) represent a significant part of the bioactive compounds obtained during the extraction of biomasses, and they are widely known for their antioxidant potential,^4^ besides presenting antibacterial activity.^1^ Several biomasses have been used for the extraction of PC, but yerba mate (*Ilex paraguariensis*) stands out as a plant matrix with high contents of these chemicals.

Yerba mate is a South American native plant largely cultivated in Brazil, Argentina, Paraguay, and Uruguay.^4^ Brazilian Institute of Geography and Statistics (IBGE) and the Paraguayan National Institute of Statistics (INE) have reported a significant increase in the yerba mate production in the countries, with yields of 441,840 and 211,420 t of green leaves in 2022 for Brazil and Paraguay, respectively.^5,6^ Its leaves are commonly used in the preparation of traditional beverages such as chimarrão, tererê, and mate tea,^7^ and they are rich in several bioactive compounds. Due to its rich chemical composition, the use of yerba mate as a raw material for the obtention of liquid extracts has considerably increased recently, with several papers evaluating the operation^4,8,9^ and a few industries in Brazil.

Attempting to align extraction processes to the Green Chemistry concept, based on the 12 principles proposed by Anastas and Warner,^10^ researchers have evaluated several process intensification pathways, which are approaches used to develop a more economically and environmentally efficient process than the conventional ones.^11^ Innumerable process intensification works on the extraction of PC from biomass can be found in the literature, for instance: Alara and Abdurahman^12^ evaluated the microwave-assisted extraction of PC from *Hibiscus sabdariffa*; Alexandre and collaborators^13^ used microwave as a pretreatment for the extraction of PC from *Arbutus unedo*; and Grillo and collaborators^14^ promoted an ultrasound-assisted extraction of PC from grape residues.

Specifically, for the yerba mate, two works relate the use of intensified strategies for the extraction of PC: López and collaborators^4^ intensified the extraction of PC using ultrasound-assisted extraction, while Rodriguez and collaborators^9^ proposed the use of pressurized liquid for the extraction of PC. However, the use of multistage batch extraction processes for the obtention of PC from yerba mate is an alternative that has not been evaluated so far. Such an approach is widely used in an industrial reactor design and is known for significantly decreasing the required equipment volume and operation time.

Hence, the current paper aims to promote process intensification on the extraction of PC from yerba mate, evaluating the use of conventional single-stage and intensified multistage extraction processes. Single-stage and two- and four-stage processes were evaluated, and techno-economic and environmental analyses were performed to evaluate the effect of such process intensification.

## Materials and Methods

### Materials

Yerba mate leaves were kindly donated by the Brazilian company Baldo (São Mateus do Sul, Paraná, Brazil). The sample was received after the conventional yerba mate drying process (*sapeco*), and it was kept at room temperature (18–25 °C) in a paper bag in the absence of light. Prior to the extraction studies, the sample was ground and sieved (48 mesh), resulting in the yerba mate powder utilized in this extraction study.

### Preliminary Extraction Analysis

Prior to the intensification study, a statistical analysis was performed to determine the effect of temperature (50–70 °C) and solid–liquid ratio (0.5–1.5 g yerba mate 100 mL^–1^ water) on the extraction of PC from yerba mate powder, following the experimental design reported in Table S1 (2^2^ factorial design with three independent central-point replicates). The extraction was conducted in batch mode in a Dubnoff water bath (NT 232, Novatecnica, Brazil) with temperature and agitation controls for 30 min. After the extraction time, the suspension was filtered with a qualitative paper filter (Qualy, J. Prolab, Brazil), and the extract was characterized for its PC content.

Additionally, the relative extraction efficiency (REE) was calculated for each run, defined as the ratio between the mass of extracted PC and the mass of yerba mate leaves used in the experiment (eq S1).

### Kinetic Study of the Single-Stage Batch Extraction

The effect of temperature and solid–liquid ratio on the extraction of PC from the yerba mate leaves was also evaluated on kinetic aspects in two univariate analyses, as presented in Table S2. Additionally, the kinetics of the best extraction condition determined according to the statistical analysis previously performed was also determined.

The kinetic analyses were conducted in a jacketed extraction vessel (inner diameter of 15 cm and height of 28 cm) with 2 L of water, and the mass of yerba mate was calculated according to the desired solid–liquid ratio. The yerba mate powder was placed onto nonwoven fabric tea bags, which were submerged in water during the extraction cycle. Temperature was controlled with a thermostatic bath with external circulation (Vivo RT4, Germany), and the agitation was maintained constant at approximately 300 rpm with a mechanical stirrer (IKA RW 20, Brazil). Samples (approximately 2 mL) were collected periodically for 5 h, and the content of the PC was determined.

Three conventional kinetic models (eqs 1,2,3, Table 1) were fitted to the experimental data on the runs conducted under different temperatures. The models were selected for their known efficiency in representing the extraction of bioactive compounds from biomasses.^8,15^

**Table 1:** Kinetic Models Fitted to the Experimental Data on the Extraction of PC from Yerba Mate Leaves under Different Temperatures

model	equation	
Pseudo-first-order	1 $C\left(t\right)=C_{eq}\left(1-\exp\left(-k_{1}t\right)\right)$	1
Pseudo-second-order	2 $C\left(t\right)=\frac{C_{eq}^{2}k_{2}t}{1+C_{eq}k_{2}t}$	2
Peleg	3 $C\left(t\right)=\frac{t}{k_{3}+k_{4}t}$	3

where *C*(*t*) (mg GAE L^–1^) is the concentration of PC in time *t* (min), *C*_eq_ (mg GAE L^–1^) is the equilibrium concentration of PC, *k*_1_ (min^–1^) is the kinetic parameter on the pseudo-first-order model, *k*_2_ (L mg^–1^ GAE min^–1^) is the kinetic parameter on the pseudo-second-order model, and *k*_3_ (min L mg^–1^ GAE) and *k*_4_ (L mg^–1^ GAE) are the kinetic parameters on the Peleg model.

Model fitting was conducted by minimizing the objective function of the mean relative error (MRE, eq S2). The model that best represented the kinetics of the extraction of PC from the yerba mate leaves was selected as the one that presented the lowest average MRE for the runs conducted under different temperatures (runs 1–3, Table S2), and it was fitted to the results obtained on the other runs.

### Study of the Multistage Batch Extraction

To intensify the extraction of PC from the yerba mate leaves, a multistage batch extraction was proposed, as presented in Figure 1.

**Figure 1:**
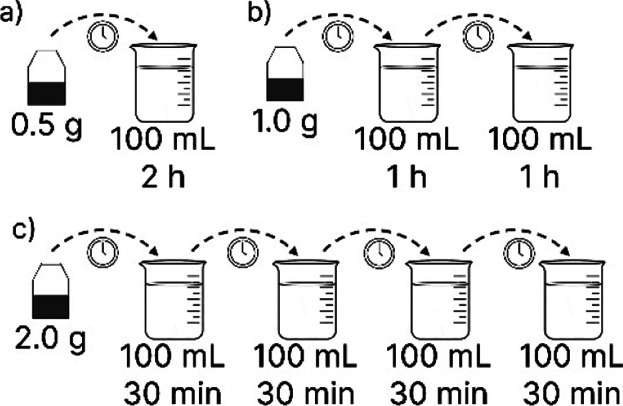
Scheme of the conventional (a), two-stage (b), and four-stage (c) extraction of PC from yerba mate.

The extraction condition that resulted in the highest REE, previously determined in the statistical analysis, was set as the global extraction condition (70 °C, 0.5 g 100 mL^–1^), and the total extraction time of 2 h was set, considering the kinetic study performed. Two intensified scenarios were evaluated: two- and four-stage batch extraction scenarios. The total time of the extraction cycle (2 h) was divided equally according to the number of stages of each scenario. Each stage was conducted with a water volume of 100 mL, and the amount of yerba mate leaves was calculated according to the solid–liquid ratio. Table 2 presents the parameters of each scenario evaluated.

**Table 2:** Extraction Parameters of Each Scenario Evaluated in the Multistep Extraction of PC from the Yerba Mate Leaves

scenario	conventional^a^	two-stage	four-stage
number of stages	1	2	4
volume per stage (mL)	100	100	100
total volume (mL)	100	200	400
mass of yerba mate leaves (g)	0.5	1.0	2.0
global solid–liquid ratio (g 100 mL^–1^)	0.5	0.5	0.5
time per stage (min)	120	60	30
total time (min)	120	120	120

a Conventional single-stage extraction scenario.

For the multistep scenarios, the calculated amount of yerba mate leaves was placed in tea bags, which were tightly closed. The tea bag was submerged in water in the extraction vessel, and after the desired contact time, it was retrieved from the vessel. The excess water in the tea bag was gently removed with a filter paper, and then the tea bag was submerged in water in the next extraction vessel.

The multistep study was conducted in jacketed glass extraction vessels. The temperature was controlled with a thermostatic bath with an external circulation. The extraction mixture was agitated with a magnetic stirrer (a magnetic bar of 2 cm length). The extract collected in each extraction step was submitted to the analysis of the content of PC, and the concentration of the mixture of the individual extracts obtained in the different extraction stages was calculated considering the mass balance described in eq 4:

4 $$C_{mix}=\frac{\sum C_{i}V_{i}}{\sum V_{i}}$$

where *C*_mix_ (mg GAE L^–1^) is the concentration of the mixture, and *C*_*i*_ (mg GAE L^–1^) and *V*_*i*_ (100 mL) are the concentration and volume of the individual extraction stages, respectively.

### Determination of the Content of Phenolic Compounds

The content of PC was determined according to ref 16. The extracts were submitted to the reaction with the Folin-Ciocalteu reagent for 2 h at room temperature (25 °C) in the absence of light. After the reaction time, the concentration was determined with a UV–vis spectrophotometer (UV-1800, Shimadzu) at 760 nm, using gallic acid as a standard for the calibration curve. The calibration curve utilized in the PC quantification is presented in the Supporting Information.

### Techno-Economic Analysis

To determine the impact of the multistage batch extraction scenarios on economic and environmental aspects, a simulation of an extraction plant was conducted. Two extraction cycles per day were considered, with the processing of 25.0 kg of yerba mate a day. The scenarios were evaluated considering the concept designs presented in Figure 2.

**Figure 2:**
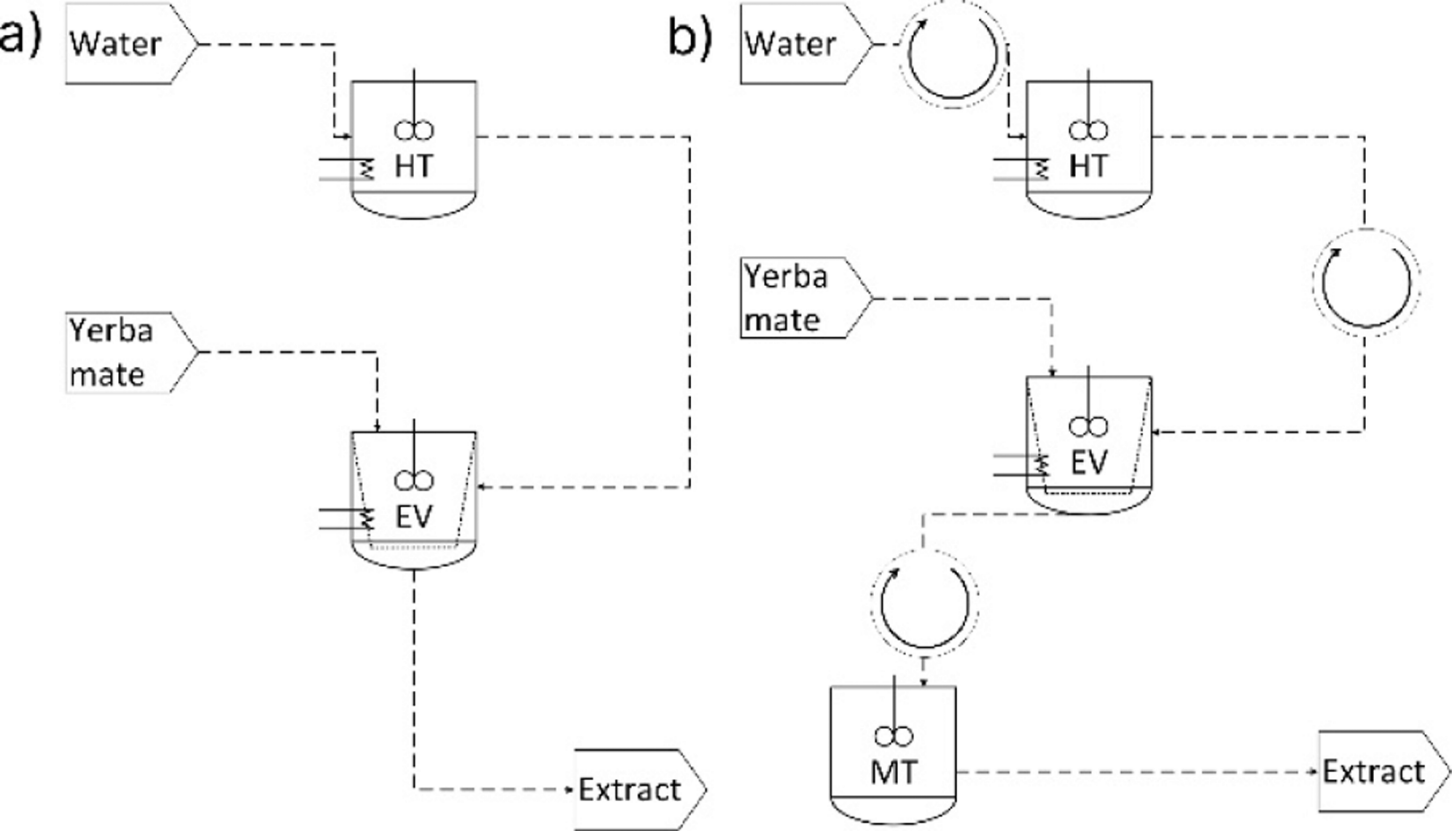
Engineering flowchart of the conventional (a) and intensified multistep (b) batch extraction of PC from yerba mate. Circular arrows indicate streams that recur at each step of the multistep extraction scenarios.

The conventional scenario consists of a heating tank (HT), used to heat water to the desired temperature before solid–liquid extraction (70 °C), and an extraction vessel (EV), from which the extract is retrieved after 2 h of extraction. The multistage scenarios consist of a HT, an extraction vessel (EV), and a mixing tank (MT), at which the extracts obtained at each stage are mixed for the entire cycle, resulting in the final extract.

Importantly, the engineering strategy adopted in this work does not involve using multiple extraction vessels (EV) for the multistage operation. Instead, a single EV is employed throughout the entire process. The EV is fed at a single moment with the yerba mate biomass, and the solvent is sequentially loaded and unloaded from the EV during the different stages, as illustrated in the Gantt chart (Figure S2).

To enable the proposed process intensification, the EV is designed with two concentric components: an outer sealed shell responsible for containing the solvent during operation and an inner removable mesh basket, which holds the solid biomass and allows the easy separation of the solid–liquid mixture at the end of each stage. This configuration ensures that the multistage extraction can be carried out in the same equipment without the need for multiple vessels.

The cycle operation time was considered equal to 3.5–4 h, which consists of the sum of the loading, extraction, and unloading times. The Gantt chart is presented in Figure S1 for the daily plant operation for the different extraction scenarios.

The techno-economic analysis was divided into three main parts: the equipment sizing and pricing, the estimation of energy consumption, and the global economic analysis, in which the Total Capital Investment (TCI) and Total Production Costs (TPC) are estimated.^17,18^ The considerations and estimation parameters of each part are described separately below.

### Equipment Sizing and Pricing

For the comparative techno-economic analysis, only the three main equipment types were considered in equipment cost: the HT, EV, and MT, and they were sized as described next. The equipment pricing was estimated considering the six-tenths rule (eq S3), and the cost and capacity of the reference equipment were retrieved from.^19^

All tanks (HT, EV, and MT) were sized considering a void fraction of 0.2 in terms of volume, considering a cylindrical shaped equipment with a height-to-diameter ratio of 1.5, which is a conventional value according to the experts,^20^ and the physical properties of the yerba mate particles were retrieved from.^21^ The sizing of each piece of equipment is completely presented in the Supporting Information.

### Estimation of Energy Consumption

An energy consumption analysis was conducted considering the energy consumption for the: (i) heating of water from room temperature (assumed as 25 °C) to the desired extraction temperature (70 °C); (ii) maintenance of the extraction temperature in the extraction vessel; and (iii) agitation of the mixtures in the HT, extraction vessel, and MT. The estimation of the energy consumption of each consideration, determined according to,^22,23^ is extensively presented in the Supporting Information.

### Global Economic Analysis

Once the equipment costs and energy consumption were determined, global economic analysis was performed according to^17,18^ using the software Microsoft Excel (v. 2024), The equations used for the estimation of each parameter are presented in Table S8.

For the estimation of the TPC, the following assumptions were considered:

1. Worked days per year: 250 days year^–1^;
2. The average roasted yerba mate leaves cost of 5.49 US$ kg^–1^;^17^
3. The average water cost in Brazil of 4.87 US$ m^–3^;
4. The average energy cost in Brazil of 8.73 × 10^–10^ US$ J^–1^.

The equations used in the estimation of the TPC are presented in Table S9.

### Sensitivity Analysis

Sensitivity analysis was conducted to identify the key factors that significantly affect the costs of the production of yerba mate extracts under different extraction scenarios. The effect of the costs of the HT, extraction vessel, MT, raw material, electricity, and labor was evaluated on the TCI and on the TPC by varying each factor at ± 20%, while maintaining the other factors constant, as reported by^24,25^

### Environmental Analysis

Finally, the different extraction scenarios were evaluated on environmental aspects, considering the generation of CO_2_ to produce the required energy for each scenario. Therefore, three energy production systems were considered: (i) the production of energy in plants using oil, with an average CO_2_ emission of 270 g CO_2_ kWh^–1^;^26^ (ii) the production of energy with the use of biofuels, which generates approximately 250 g CO_2_ kWh^–1^ (biodiesel);^26^ and (iii) the production of energy in hydroelectric plants, at which there is no direct CO_2_ production but an average indirect production of 4 g CO_2_ kWh^–1^.^27^ These production systems were chosen because they represent a significant part of energy production in Brazil.^28^

## Results and Discussion

### Preliminary Extraction Analysis

Several parameters present a significant influence over the extraction of biomolecules from biomasses, and the temperature and solid–liquid ratio usually represent two of the major factors. In this context, the influence of these variables on the extraction of PC from yerba mate has been evaluated, and the results on the concentration and REE of these compounds are presented in Figure 3 and Table S10. According to the contour line graph (Figure 3a), both the increase in the temperature and in the solid–liquid ratio result in the increase of the concentration of PC in the extract obtained after 30 min of extraction, with the highest concentration obtained at the highest temperature with the highest solid–liquid ratio (70 °C and 1.5 g 100 mL^–1^). The Pareto chart (Figure 3b) indicates that both temperature and solid–liquid ratio showed an impact on the concentration of the extract (*p* ≤ 0.05), with the solid–liquid ratio being the most significant parameter.

**Figure 3:**
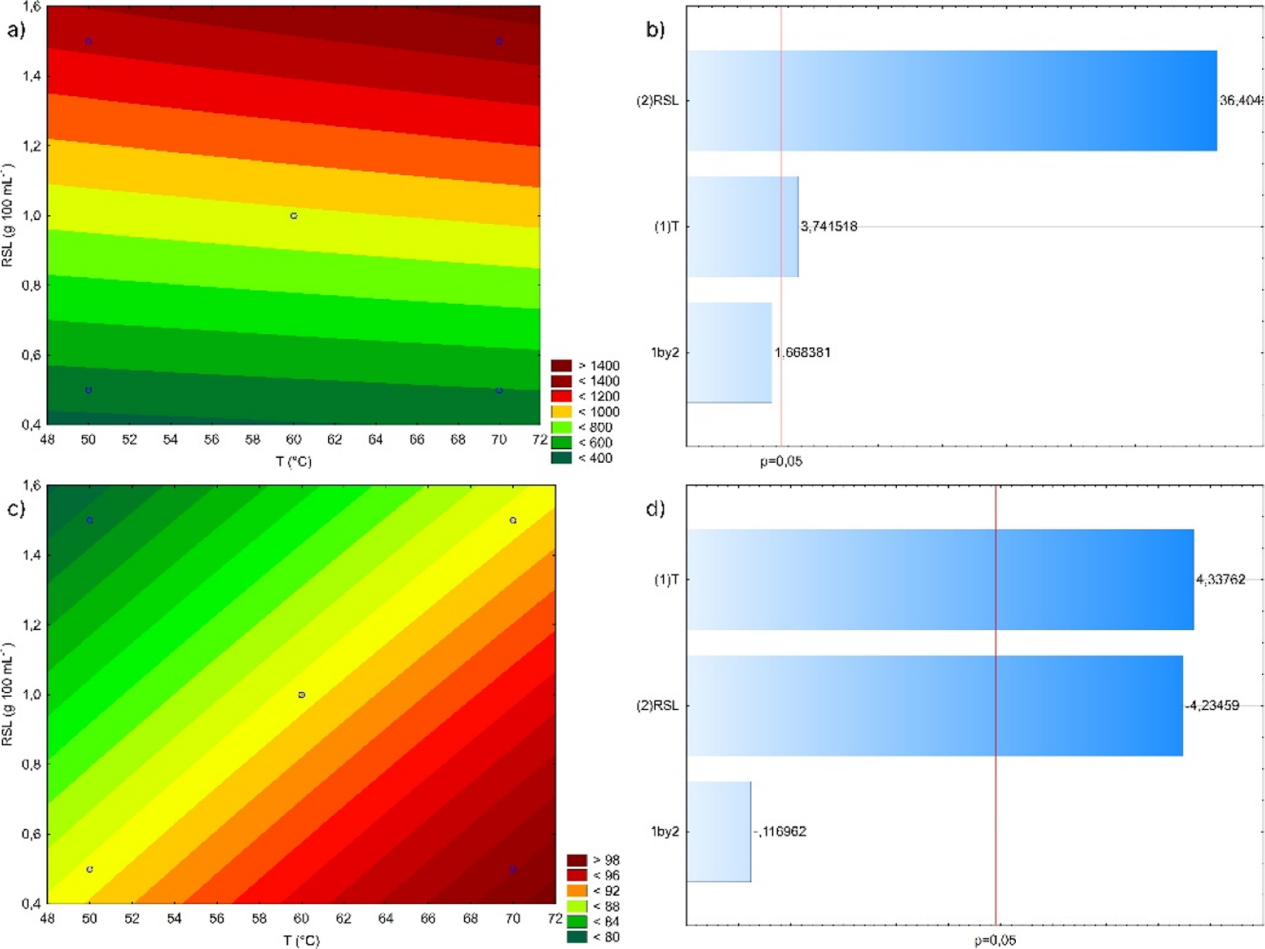
Contour line graph and Pareto chart of the influence of temperature and solid–liquid ratio on the concentration of PC (a,b) and on the REE of PC (c,d) from yerba mate onto water after 30 min of batch extraction. Contour line graphs and Pareto charts were generated in the Statistica software (v.10).

Regarding the REE, Figure 3c indicates that the highest REE is obtained in the extraction conducted under the highest temperature with the lowest solid/liquid ratio (70 °C and 0.5 g 100 mL^–1^). The Pareto chart (Figure 3d) indicates that both parameters have a significant influence on the REE with similar influence levels. Although the REE does not provide precise information about the degree of lixiviation of PC from the biomass, as it is not calculated based on the total mass of PC present in the yerba mate, the parameter remains a valuable comparative tool to identify the condition at which more PC are extracted from the solid matrix. The extraction condition with the highest REE also reflects the scenario with the lowest residual PC content in the lignocellulosic material after the extraction process, consequently resulting in the scenario with the lowest PC waste.

The selection of the best extraction condition, according to Figure 3, can be carried out considering two different objectives: the condition that results in the extract with the highest concentration of PC (70 °C and 1.5 g 100 mL^–1^) or the condition that promotes the highest extraction of the PC from the biomass (70 °C and 0.5 g 100 mL^–1^). At this point, considering the potential of developing a biorefinery plant from yerba mate with the complete use of the plant, the best extraction condition was chosen as the one that results in the highest extraction efficiency.

### Kinetics of the Single-Stage Batch Extraction

As previously observed, both the temperature and the solid/liquid ratio present a significant influence on the extraction of PC from yerba mate. The influence of these parameters was then evaluated in extraction kinetics, in two univariate analyses, and the results are presented in Figures 4 and 5. The kinetic assay is an important study of the extraction of bioactive compounds from biomass, as it provides essential information on the time required for the process. Specifically, for the intensification study proposed in this work, the kinetic assay can be used to set the global time of extraction, which will be divided according to the number of extraction stages used.

**Figure 4:**
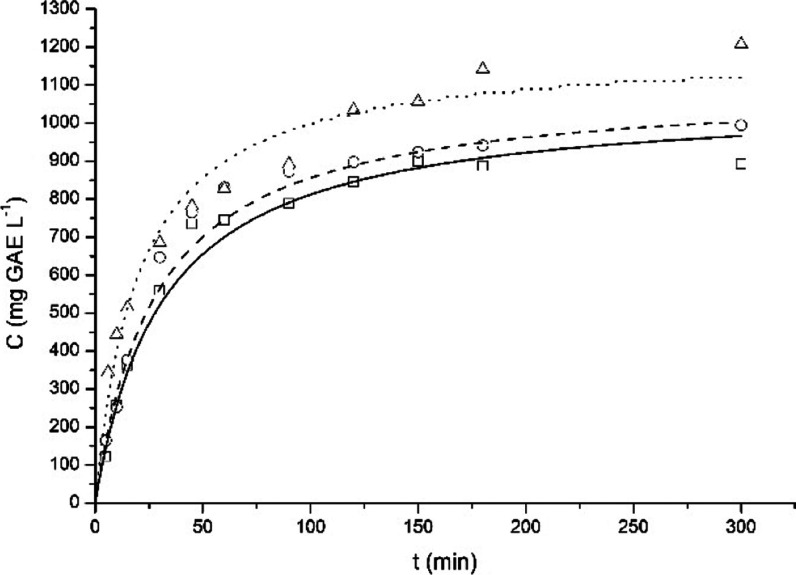
Kinetics of the extraction of PC from yerba mate at different temperatures: 50 (squares), 60 (circles), and 70 °C (triangles), and Peleg’s kinetic model fitted to the experimental data: 50 (straight line), 60 (dashed line), and 70 °C (dotted line). Data obtained with a solid–liquid ratio of 1.0 g 100 mL^–1^ and a rotation speed of 300 rpm.

**Figure 5:**
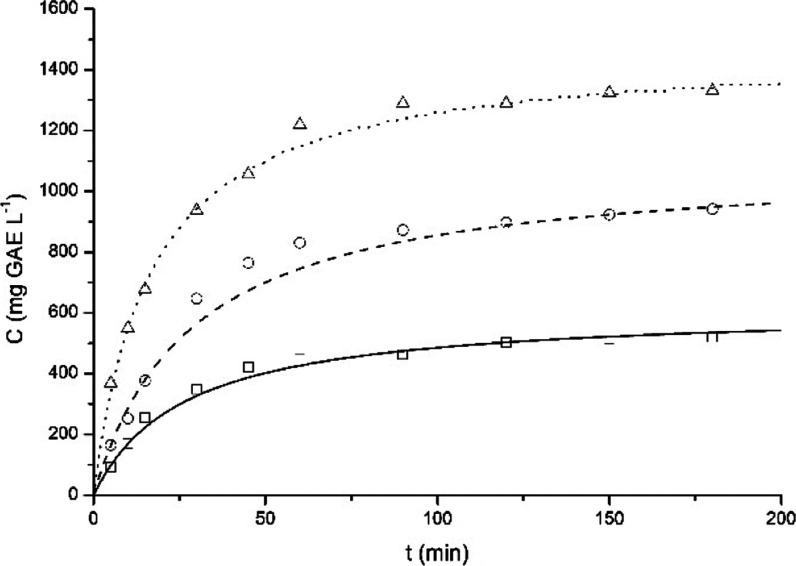
Kinetics on the extraction of PC from yerba mate with different solid–liquid ratios: 0.5 (squares), 1.0 (circles), and 1.5 g 100 mL^–1^ (triangles), and Peleg’s kinetic model fitted to the experimental data: 0.5 (straight line), 1.0 (dashed line), and 1.5 g 100 mL^–1^ (dotted line). Data obtained at 60 °C with a rotation speed of 300 rpm.

The temperature significantly influences the extraction kinetics (Figure 4), especially the initial extraction rate: the increase in the temperature results in an expressive increase of the initial extraction rate (Table 3), which can be associated with the lower viscosity of water and the higher diffusivity coefficient of the solute/solvent system.^15^ An equilibrium-like state is achieved during the extraction, and the concentration of PC at 3 h is also affected by the temperature: higher temperatures result in higher PC contents. Such results are similar to the ones obtained by Gerke and collaborators,^8^ who also evaluated the batch extraction of PC from yerba mate.

**Table 3:** Initial Phenolic Compounds’ Extraction Rate and Fitted Parameters on the Kinetics Obtained under Different Extraction Temperatures (50–70 °C)

extraction temperature (°C)	50	60	70
phenolic compounds’ extraction rate at the initial 15 min (mg GAE L^–1^ min^–1^)	24.1	25.1	34.3
pseudo-first-**order**			
*C*_eq_ (mg GAE L^–1^)	890	931	1039
*k*_1_ (min^–1^)	3.31 × 10^–2^	3.71 × 10^–2^	4.56 × 10^–2^
MRE	0.035	0.038	0.12
average MRE	0.063		
pseudo-second-**order**			
*C*_eq_ (mg GAE L^–1^)	1068	1103	1047
*k*_2_ (L mg^–1^ GAE min^–1^)	2.97 × 10^–5^	3.18 × 10^–5^	7.04 × 10^–5^
MRE	0.058	0.051	0.070
average MRE	0.060		
**Peleg**			
*k*_3_ (min L mg^–1^ GAE)	2.95 × 10^–2^	2.58 × 10^–2^	1.66 × 10^–2^
*k*_4_ (L mg^–1^ GAE)	9.37 × 10^–4^	9.10 × 10^–4^	8.36 × 10^–4^
MRE	0.058	0.051	0.066
average MRE	0.058		

Three mathematical models were fitted to the experimental data, and the fitting error and the fitted parameters are reported in Table 3.

The mathematical model with the lowest average MRE was Peleg’s model, which was assumed to be the best model to describe the extraction kinetics of PC from yerba mate, similar to the results obtained by Gerke and collaborators.^8^

The solid–liquid ratio also resulted in a significant influence on the extraction of PC from yerba mate in both the initial extraction rate and the final concentration of PC (Figure 5), as reported in Table 4. Peleg’s model was also fitted to the experimental data of the extraction kinetics with different solid–liquid ratios, and the fitted parameters are reported in Table 4.

**Table 4:** Initial Phenolic Compounds’ Extraction Rate, Concentration of Phenolic Compounds, and Relative Extraction Efficiency after 3 h of Extraction, and Fitted Parameters on the Kinetics Obtained with Different Solid–Liquid Ratios (0.5–1.5 g 100 mL^–1^)

solid–liquid ratio (g 100 mL^–1^)	0.5	1.0	1.5
phenolic compounds’ extraction rate at the initial 15 min (mg GAE L^–1^ min^–1^)	17.0	25.1	45.2
concentration of phenolic compounds at 3 h of extraction (mg GAE L^–1^)	520	942	1331
relative extraction efficiency of phenolic compounds at 3 h of extraction (mg GAE g^–1^ yerba mate)	104	94.2	88.7
Peleg			
*k*_3_ (min L mg^–1^ GAE)	4.29 × 10^–2^	2.58 × 10^–2^	1.14 × 10^–2^
*k*_4_ (L mg^–1^ GAE)	1.63 × 10^–3^	9.10 × 10^–4^	6.81 × 10^–4^
MRE	0.043	0.051	0.027
average MRE	0.040		

Similar to the observation in the preliminary extraction study, the use of a higher solid–liquid ratio resulted in an increase in the concentration of PC in the extract; however, it also resulted in a decrease in the REE.

Finally, the kinetics of the best extraction conditions (70 °C and 0.5 g 100 mL^–1^), selected according to the preliminary extraction study, was evaluated, and the result is presented in Figure 6.

**Figure 6:**
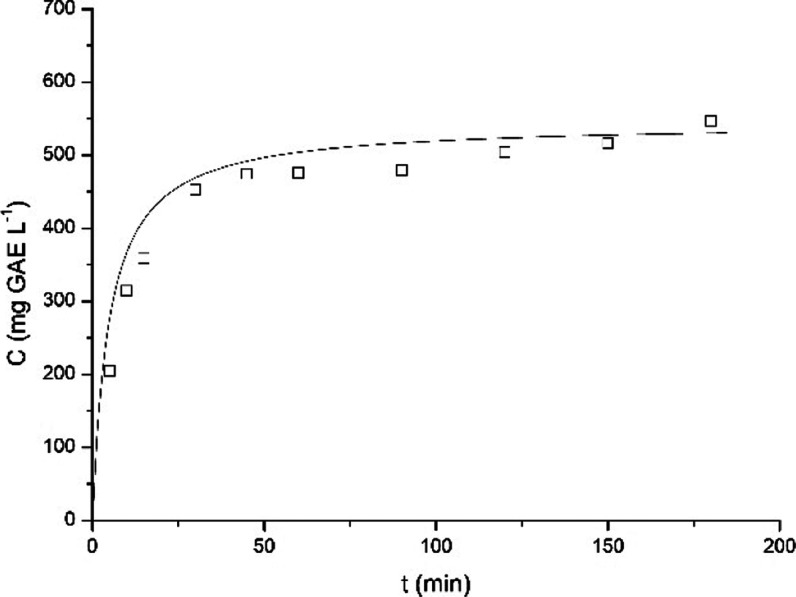
Kinetics on the extraction of PC from yerba mate at the best extraction condition according to the preliminary extraction study, with Peleg’s kinetic model fitted to the experimental data: 70 °C, 0.5 g 100 mL^–1^ and a rotation speed of 300 rpm.

Table S11 presents the parameters of Peleg’s model fitted to the experimental data of the best extraction condition.

### Multistage Batch Extraction

Attempting to promote an intensification of the extraction process, a multistage batch extraction was evaluated. For this reason, the best extraction condition previously observed was considered (70 °C and 0.5 g 100 mL^–1^), and an extraction time of 2 h was set, according to the extraction kinetics (Figure 6). Two multistage extraction scenarios were evaluated: two-stage and four-stage extraction. The total water volume, calculated according to the solid–liquid ratio of 0.5 g 100 mL^–1^ and the mass of yerba mate used in the extraction, and the total extraction time of 2 h were divided equally for the different stages. The results on the concentrations of PC for each stage are presented in Table 5.

**Table 5:** Concentration of PC in the Extract of Yerba Mate Obtained in Each Stage of Extraction for the Different Extraction Scenarios^a^

scenario	conventional	two-stage	four-stage
concentration of first stage^1^	503.8 ± 7.4	976.6 ± 16.0	1715.3 ± 9.0
concentration of second stage^1^	-	76.3 ± 6.1	221.2 ± 2.0
concentration of third stage^1^	-	-	28.3 ± 1.8
concentration of fourth stage^1^	-	-	7.4 ± 0.4
concentration of the mixture^1,2^	503.8 ± 7.4	526.5 ± 11.1	493.0 ± 2.9

a Results expressed as mean ± standard deviation. Concentration expressed in mg GAE L^–1^ (^1^), and concentration of the mixture calculated according to a mass balance of the concentrations of the individual extraction stages (^2^).

According to the results reported in Table 5, the concentration of the extract obtained in the first extraction stage increases with the use of multiple stages, which can be associated with the individual solid–liquid ratio per stage in the different extraction scenarios. Maintaining the global solid–liquid ratio of 0.5 g 100 mL^–1^ and dividing the water volume into separate stages result in the increase of the individual solid–liquid ratio per stage: individual solid–liquid ratios per stage of 0.5, 1.0, and 2.0 g 100 mL^–1^ are achieved for the conventional, two-stage, and four-stage scenarios, respectively.

When considering the mixing of the extracts obtained in the individual stages into a single extract, a mass balance is applied to determine the final concentration of PC, which is presented in Table 5. The results indicate that there are statistical differences in the concentration of PC in the final extracts obtained under the different extraction scenarios, but the effective difference can be negligible, suggesting that the two multistage extraction scenarios can be employed for the obtention of a product with a similar antioxidant potential to the conventional extraction scenario.

Regarding the REE of the three evaluated extraction scenarios (Table S12), the use of the intensified multistage extraction process results in the decrease of the REE of the individual stages, which is corroborated by the result presented in Figure 3C: when operating with a higher individual solid/liquid ratio, a lower REE is expected. The global REE of the process, however, is similar for the three extraction scenarios (98.6–105.3 mg GAE g^–1^ yerba mate), which, in accordance to the similar concentration of the mixture (493.0–526.5 mg GAE L^–1^), highlights the exchangeability of the three approaches. Hence, the selection of the best extraction scenario must be made considering techno-economic and environmental aspects, which are presented ahead.

### Techno-Economic and Environmental Analysis

To determine the efficiency of the process intensification proposed in the multistage extraction approach, techno-economic and environmental analyses were conducted. A processing of 25 kg yerba mate day^–1^ was considered, which results in a production of 5.0 m^3^ extract day^–1^, equivalent to 1250 m^3^ year^–1^. Initially, the equipment of each extraction scenario was sized, considering a mass balance for each unitary operation and empiric correlations, presented in the Supporting Information. The use of the intensified multistage extraction process resulted in a considerable decrease in the required size for the HT and extraction vessel (EV) equipment: for instance, a 3.14 m^3^ EV is required for the conventional process, whereas 1.57 and 0.792 m^3^ EVs are required for the two- and four-stage intensified extraction processes, respectively.

The observed decrease in the required size for HT and EV is also reflected in the equipment cost (Table S13). The equipment cost consists of the sum of the costs of the HT, EV, and MT. The multistage intensified extraction scenarios result in an equipment cost considerably lower than the one required for the conventional scenario, although the intensified scenarios require an extra equipment (MT). Such a result is associated with the fact that, even though an extra equipment is required, MT is a cheaper equipment than HT and EV, as it is a simple vessel with no heating or temperature control.

The raw material cost (Table S14) is equal for all three scenarios, as it was considered to be the same amount of yerba mate processed per day, and it was determined considering the price of the water used in the extraction and the yerba mate. Labor costs (Table S15) were also considered equal for the three scenarios: three plant workers and one engineer.

Table S16 presents the energy consumption on the main equipment of the extraction process for the different scenarios and the annual cost for the energy consumption. According to Table S16, the step with the major energy consumption during the extraction of PC from the yerba mate is the heating of water from room temperature to extraction temperature (50.2 GJ cycle^–1^). The value obtained for each scenario is the same, for there is no difference in the total amount of water used in the extraction process, in the heat capacity of the solvent, or in the initial and final temperatures.

The energy required for maintaining the temperature on the EV and for the agitation of the mixtures in the HT, EV, and MT, however, varies according to the volume of the mixture and the operation time. The decrease in the required size for the HT and the EV in the multistage extraction scenarios also affects the energy consumption of the process, and it results in considerable savings in the energy consumption (Table S16), which represents an annual energy saving of 431.6–681.1 MJ year^–1^.

Finally, global economic analysis was performed, and TCI (Table S17) and Total Production Cost (Table S18) were determined for each extraction scenario.

Table 6 summarizes the main results of the techno-economic and environmental analyses of the different extraction scenarios. The use of the intensified multistep extraction process results in a lower equipment cost, specifically due to the lower capacity needed for the HT and extraction vessel. Lower equipment costs result in a lower TCI. Consequently, the intensified multistep extraction scenarios also present a considerably lower TCI. The four-step extraction scenario, for instance, presents a TCI saving of up to 31.0% of the TCI of the conventional extraction scenario.

**Table 6:** Main Results on the Global Economic and Environmental Analyses of the Different Scenarios of Extraction of Phenolic Compounds from Yerba Mate^a^

scenario	conventional	two-stage	four-stage
**economic aspects**			
equipment cost (US$)	6493	5929	4476
energy cost (US$ year^–1^)	21,909	21,909	21,908
total capital investment (US$)	17,082	15,600	11,777
total production cost (US$ year^–1^)	129,587	129,231	128,311
**environmental aspects**			
energy saving^1^ (MJ year^–1^)	-	431.6	681.1
CO_2_ emission saving—oil system (kg year^–1^)	-	32.4	51.1
CO_2_ emission saving–biofuel system (kg year^–1^)	-	30.0	47.3
CO_2_ emission saving–hydroelectric system (kg year^–1^)	-	0.480	0.757

a Energy saving is calculated as the difference between the energy consumption of the evaluated scenario and the energy consumption of the conventional scenario (^1^).

Regarding the production costs, the raw material and labor costs are equal for all extraction scenarios. Although there is a significant energy saving in the intensified multistep extraction scenarios, when compared to the conventional one, such saving is not significant in economic aspects, and the energy cost is not significantly different for the three scenarios. Consequently, the total production cost is similar for the different extraction scenarios.

One-way sensitivity analysis was performed to identify the influence of selected parameters in the range of ±20% on the TCI and TPC of the three extraction scenarios, and the results are presented in Figures 7 and 8, with the most influential parameter of each scenario marked with a star. From Figure 7, it was found that the most influential factor on the TCI in the conventional and in the two-stage extraction scenarios is the cost of the extraction vessel (EV), which results in a variation of ±1706 and 1126 US$ for these scenarios. For the four-stage extraction scenario, on the other hand, the most influential factor on the TCI is the MT, resulting in a variation of ±743 US$. Such results are related to the decrease in the required volume of the EV and the HT when using the intensified extraction scenarios with a constant-volume MT.

**Figure 7:**
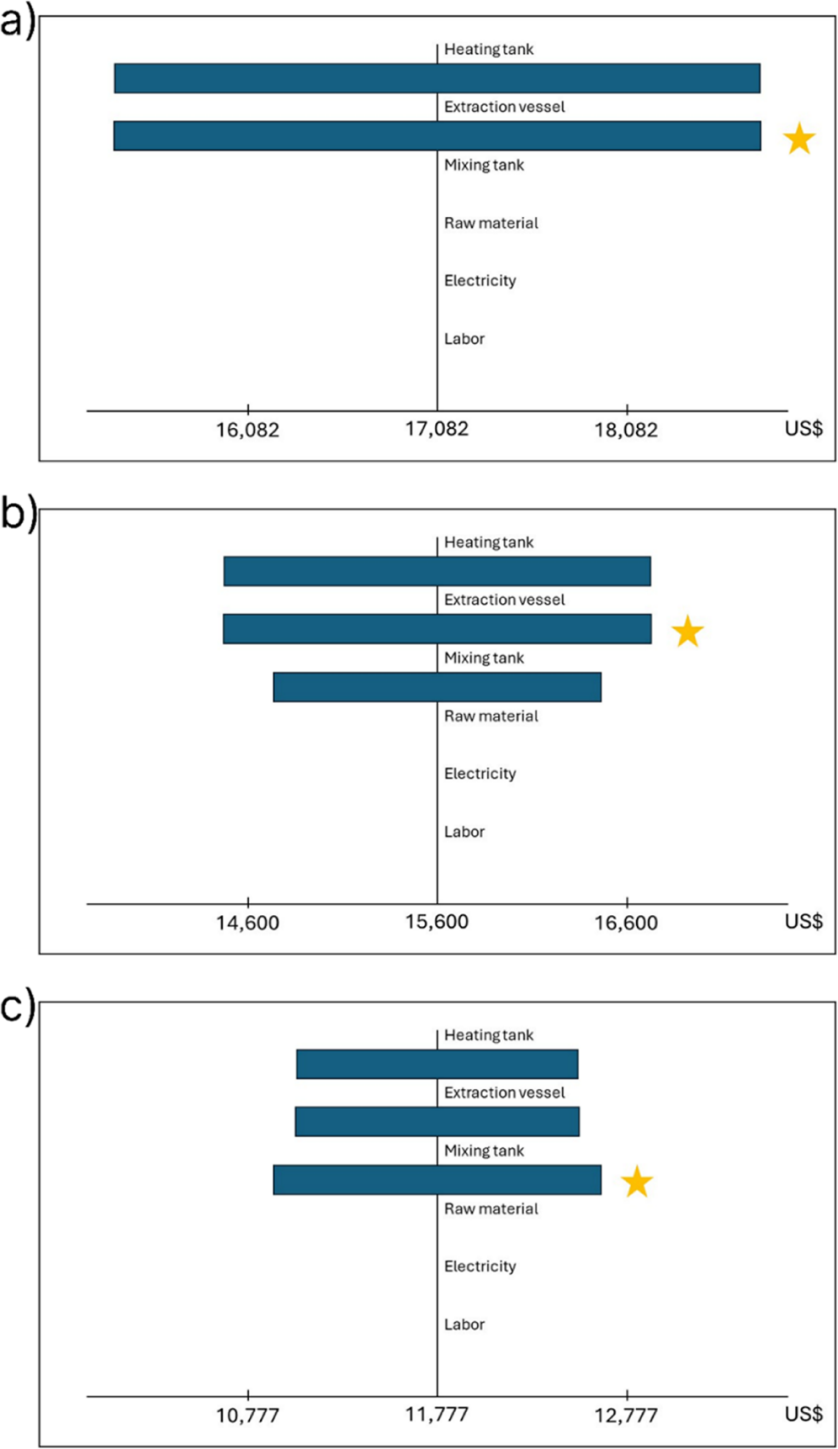
Sensitivity analysis for the TCI on the conventional (a), two-stage (b), and four-stage (c) extraction scenarios.

**Figure 8:**
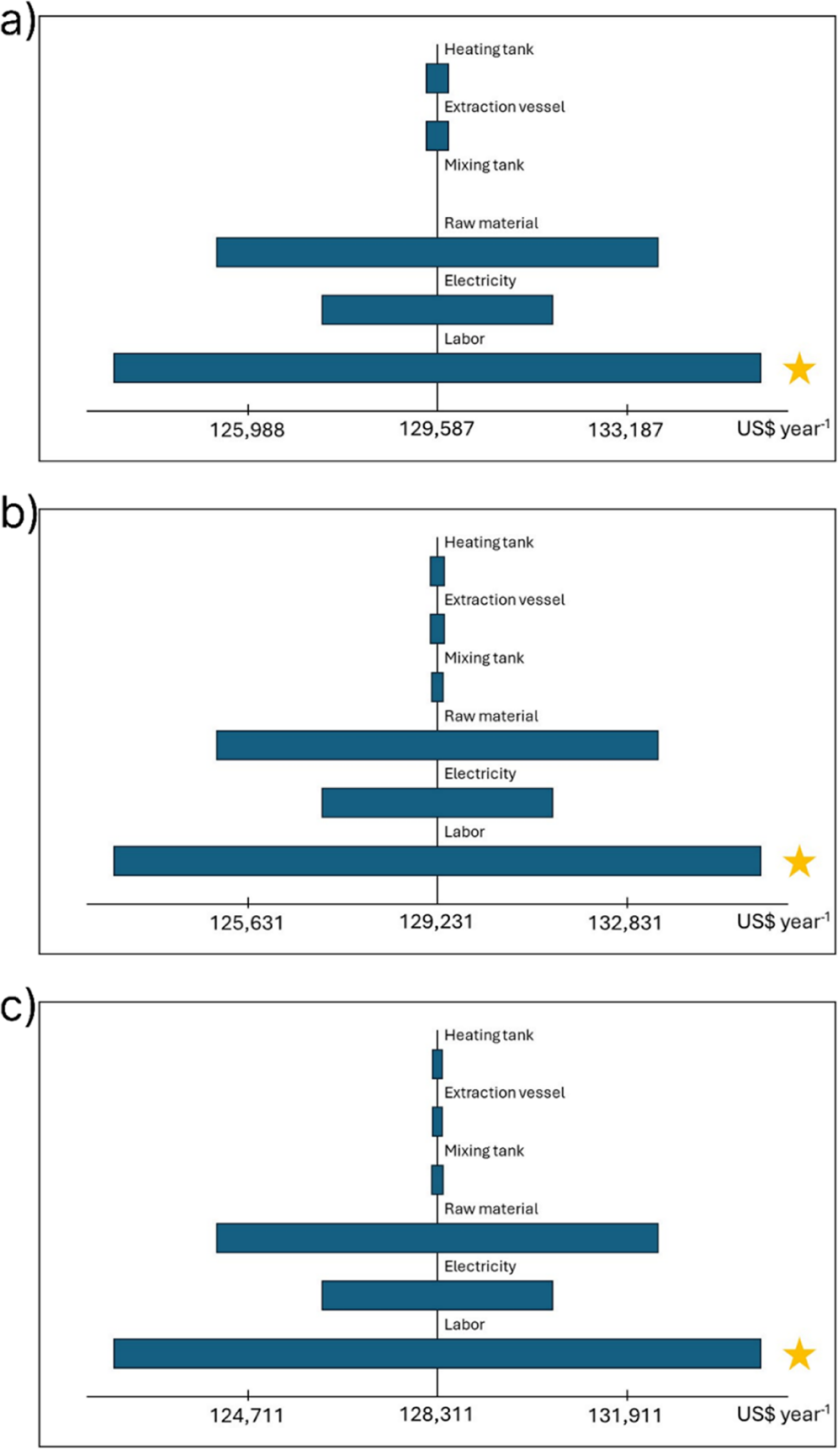
Sensitivity analysis for the TPC on the conventional (a), two-stage (b), and four-stage (c) extraction scenarios.

Regarding the one-way sensitivity analysis on the TPC, Figure 8 indicates that the most influential parameter on the TPC for the three extraction scenarios at a ±20% range is the Labor cost, which results in a variation of ±12,311 US$ year^–1^, followed by the Raw Material cost, representing a variation of ±8403 US$ year^–1^.

When considering the environmental aspects, the energy saving obtained in the intensified multistep scenario is of great importance, especially when considering the CO_2_ emission related to energy generation. In Brazil, energy is mainly generated with oil, biofuels, or hydroelectric systems (36.9, 32.9, and 11.8% of the total energy production in Brazil in 2023, respectively).^2^ The production of energy with oil or biofuels is related to high direct CO_2_ emission levels, while hydroelectric systems do not promote direct CO_2_ emissions, but they are known for indirect CO_2_ emission.^26^ At this point, the three energy production systems were considered, and the savings in the emission of CO_2_ for the different extraction scenarios are reported in Table 6. Such result highlights the importance of intensification in the environmental field, as it promotes the concepts of green chemistry proposed by Anastas and Warner,^10^ which are usually extended to the economic and engineering fields.

## Conclusions

This work presented an alternative intensified process for the extraction of PC from yerba mate. The intensified process, which consists of multiple sequential stages, results in a plant extract similar to the one obtained under the conventional batch extraction, with a considerable PC content (up to 526.6 mg GAE L^–1^ for the two-stage extraction scenario). The processes (conventional and intensified) were simulated for an industrial scale-up scenario, considering the processing of 25 kg yerba mate day^–1^, and the multistage process is estimated to promote an economic saving of up to 31.0% of the TCI required when compared to the conventional scenario. The multistage scenario also resulted in considerable environmental benefits, as it reduces significantly the energy consumption and, consequently, the generation of CO_2_ during energy generation, with a CO_2_ emission saving of up to 51.1 kg CO_2_ year^–1^, considering the four-stage extraction scenario with an oil-based energy production system. The proposed process intensification results in benefits in both economic and environmental aspects, justifying its implementation in industries such as plant extracts. Further investigations on the design of the extraction vessel (EV) for the multistage extraction scenarios are required, in order to develop an easy-to-operate EV that allows both the extraction and the facilitated separation of the biomass from the liquid extract.

## Supplementary Material



## Notes

Additional scale-up simulation details, including all results of the techno-economic analysis (PDF)
