# Supplementary material for: Intensified Multistep Extraction of Phenolic Compounds from Yerba Mate (Ilex paraguariensis) Leaves: A Techno-Economic and Environmental Approach
Source: ACS Omega. 2026 Feb 4;11(6):9816–25. doi: 10.1021/acsomega.5c10400 (PMC12917618; doi:10.1021/acsomega.5c10400)
Supplement: Supplementary file 1 [file ao5c10400_si_001.pdf]

SUPPORTING INFORMATION

Intensified multi-step extraction of phenolic compounds from yerba mate (*Ilex paraguariensis*) leaves: a techno-economic and environmental approach

Matheus Samponi Tucunduva Arantes [†,*], Sara Mariele Nohr de Lima [†], Giovana Gonçalves Dusi [†], Cristiane Vieira Helm [‡], Washington Luiz Esteves Magalhães [‡], Vítor Renan da Silva [†]

[†] Chemical Engineering Department, Federal University of Paraná (UFPR), Curitiba, PR, Brazil.

[‡] Embrapa Florestas, Colombo, PR, Brazil.

*Corresponding author. E-mail address: matheussamponi@ufpr.br

MATERIAL AND METHODS

Preliminary extraction analysis

Table S1. Experimental design for the statistical study of the extraction of phenolic compounds from yerba mate leaves.

Run	Temperature (°C)	Solid-Liquid Ratio (g 100 mL^{-1})
1	50	0.5
2	50	1.5
3	60	1.0
4	70	0.5
5	70	1.5

Equation S1 was used to calculate the relative extraction efficiency, where *REE* (mg GAE g^{-1} yerba mate) is the relative extraction efficiency, C_{PC} (mg GAE L^{-1}) is the concentration of phenolic compounds in the extract, V_{ext} (0.050 L) is the extract volume, and w_{YM} (g) is the amount of yerba mate used in the extraction.

$$REE = \frac{(C_{PC}\, V_{ext})}{w_{YM}} \quad \text{(S1)}$$

Kinetics study of the single-stage batch extraction

Table S2. Experimental design for the kinetics study of the extraction of phenolic compounds from yerba mate leaves.

Run	Temperature (°C)	Solid-Liquid Ratio (g 100 mL^{-1})
1	50	1.0
2	60	1.0
3	70	1.0
4	60	0.5
5	60	1.5
6[1]	70	0.5

Best extraction condition determined according to the statistical analysis previously performed ([1]).

Equation S2 was used as the objective function for the mathematical modeling of the extraction kinetics, where N is the number of experimental data, and C_{calc} and C_{exp} (mg GAE L^{-1}) are the calculated and experimental values of the concentration of phenolic compounds.

$$MRE = \frac{1}{N} \sum_{i=1}^{N} \frac{|C_{calc} - C_{exp}|}{C_{exp}} \quad \text{(S2)}$$

Determination of the content of phenolic compounds

The calibration curve used for the quantification of phenolic compounds was constructed using analytical-grade gallic acid. A stock solution of 100 mg L^{-1} was initially prepared by dissolving 10 mg of the acid in 100 mL of deionized water in a volumetric flask. This stock solution was then diluted with deionized water to obtain the calibration standards (concentrations ranging from 1.0 to 9.0 mg L^{-1}). Finally, the calibration solutions were submitted to the Folin-Ciocalteu reaction, and their absorbance was measured (760 nm). The resulting calibration curve is presented in Fig. S1.

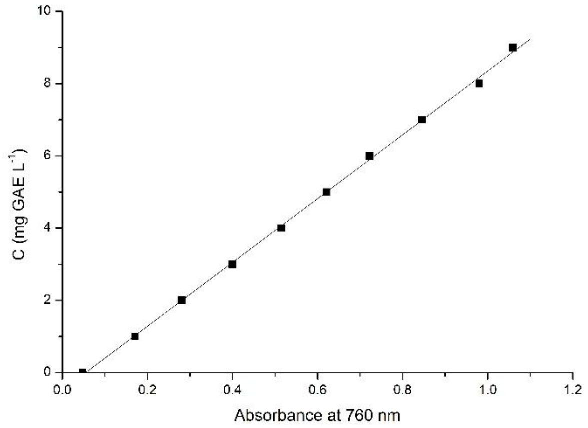

Figure S1. Calibration curve used in the quantification of phenolic compounds.

The experimental data were submitted to a linear regression using the *Solver* tool in the *Microsoft Excel*, and the parameters a and b from Eq. S3 were determined.

$$C = a\,Abs + b \quad \text{(S3)}$$

Where C (mg L^{-1}) is the concentration of gallic acid in the solution, Abs is the absorbance at 760 nm, and a (8.84) and b (-0.48 mg L^{-1}) are the fitted parameters. The fitting of the calibration curve resulted in a coefficient of determination (R^2) of 0.999.

Techno-economic analysis

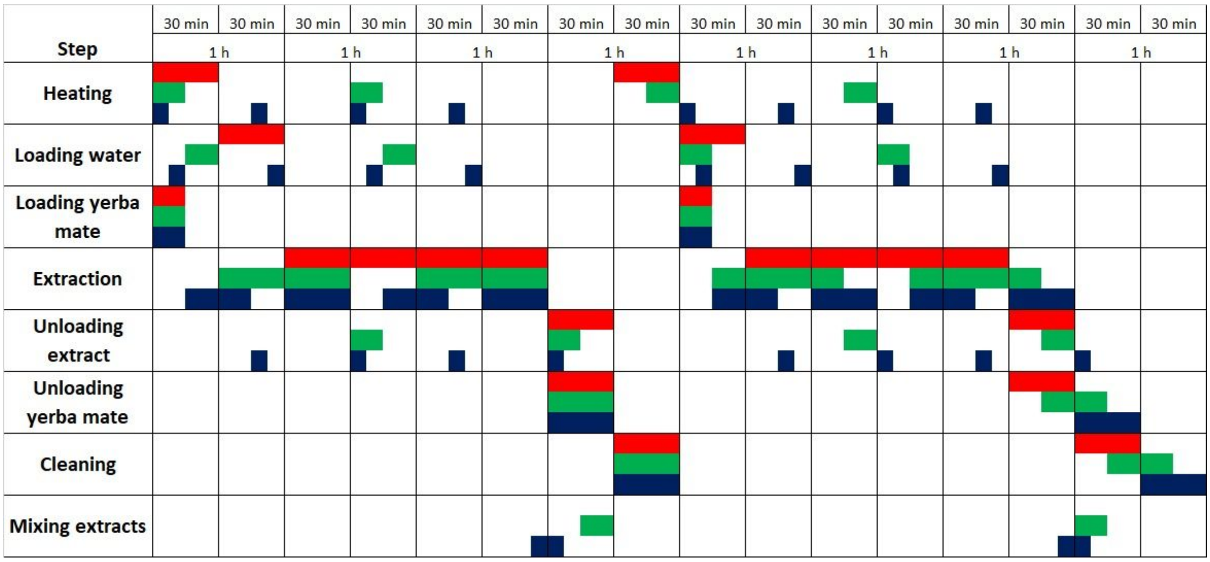

Figure S2. Gantt chart on the extraction of phenolic compounds from yerba mate under different extraction scenarios: conventional (red), and intensified two-step (green) and four-step (blue).

Equipment sizing and pricing

Equation S4 was used to estimate the cost of the equipment based on references from the literature, where C_1 and C_2 (US$ unity^{-1}) are the costs of the equipment of reference and the sized for the current simulation, respectively, Q_1 and Q_2 (m^3) are the capacities of the equipment of reference and the sized for the current simulation, respectively, and n (0.6) is the exponential term in the six-tenths rule.

$$C_2 = C_1\left(\frac{Q_2}{Q_1}\right)^n \quad \text{(S4)}$$

The reference cost and capacity used for the price estimation of the equipment are presented in Table S3. These values were retrieved from [19].

Table S3. Costs and capacity of the reference equipment.

Equipment	Reference cost (US$ unity^{-1})	Reference capacity (m^3)
Heating tank and extraction vessel[1]	1,080	0.50
Mixing tank[2]	5,000	20

The heating tank and extraction vessel consist of a tank with agitation and temperature control ([1]), while the mixing tank requires only agitation ([2]).

Heating tank sizing

Initially, the amount of yerba mate used per extraction cycle was calculated, considering the daily processing and number of cycles. The volume of water used in the extraction was calculated considering the solid-liquid ratio of 0.5 g 100 mL^{-1} (equivalent to 5 g L^{-1} or 5 kg m^{-3}). The volume of water used per stage was calculated as the ratio between the water used per cycle and the

number of stages per cycle. The volume of the HT was calculated considering the volume of water per stage and the void fraction, and its height and diameter were calculated considering the desired h/D. All parameters considered and results obtained in the sizing of the heating tank are presented in Table S4.

Table S4. Parameters and results on the heating tank sizing for the different extraction scenarios.

Scenario	Conventional	Two-stage	Four-stage
Amount of yerba mate per cycle (kg $cycle^{-1}$)	12.5	12.5	12.5
Volume of water per cycle (m^3 $cycle^{-1}$)	2.50	2.50	2.50
Number of stages	1	2	4
Volume of water per stage (m^3 $stage^{-1}$)	2.50	1.25	0.625
Void fraction	0.2	0.2	0.2
**Volume of the heating tank (m^3)**	**3.13**	**1.56**	**0.781**
h/D	1.5	1.5	1.5
Height (m)	2.08	1.65	1.31
Diameter (m)	1.38	1.10	0.872

Extraction vessel sizing

Initially, the volume of yerba mate powder processed per extraction cycle was calculated considering the amount of yerba mate per cycle and the density of the yerba mate particles (ρ_S, 1,488.1 kg m^{-3}) [21]. The mixture volume was calculated as the sum of the yerba mate volume and the volume of water per stage. Then, the volume of the EV was calculated considering the mixture volume and the void fraction, followed by the estimation of the height and diameter. All parameters considered and results obtained in the sizing of the heating tank are presented in Table S5.

Table S5. Parameters and results on the extraction vessel sizing for the different extraction scenarios.

Scenario	Conventional	Two-stage	Four-stage
Amount of yerba mate per cycle (kg $cycle^{-1}$)	12.5	12.5	12.5
Volume of yerba mate per cycle (m^3)	8.40 E-03	8.40 E-03	8.40 E-03
Volume of water per stage (m^3 $stage^{-1}$)	2.50	1.25	0.625
Volume of mixture per stage (m^3 $stage^{-1}$)	2.51	1.26	0.633
Void fraction	0.2	0.2	0.2
**Volume of the extraction vessel (m^3)**	**3.14**	**1.57**	**0.792**

h/D	1.5	1.5	1.5
Height (m)	2.08	1.65	1.31
Diameter (m)	1.39	1.10	0.876

Mixing tank sizing

The volume of the mixing tank was calculated considering the volume of water per extraction cycle and the void fraction, and the height and diameter were estimated as presented in Table S6.

Table S6. Parameters and results on the mixing tank sizing for the different extraction scenarios.

Scenario	Conventional[1]	Two-stage	Four-stage
Volume of extract per cycle (m^3 $cycle^{-1}$)	2.50	2.50	2.50
Void fraction	-	0.2	0.2
**Volume of the mixing tank (m^3)**	**-**	**3.13**	**3.13**
h/D	-	1.5	1.5
Height (m)	-	2.08	2.08
Diameter (m)	-	1.38	1.38

The conventional scenario does not require a mixing tank ([1]).

Estimation of energy consumption

Heating of the water (25 to 70 °C) in the heating tank

The energy consumption for the heating of the water from 25 to 70 °C per extraction cycle was calculated considering Eq. S5.

$$dQ = n_{stage} \frac{w_w}{MM_w} \int_{T_o}^{T_f} Cp(T)\, dT \qquad \text{(S5)}$$

Where Q (J $cycle^{-1}$) is the energy consumption per cycle, n_{stage} (stage $cycle^{-1}$) is the number of stages per cycle, w_w (kg $stage^{-1}$) is the amount of water used per stage, MM_w (18.01528 kg $kmol^{-1}$) is the molar mass of the water, retrieved from [22], *Cp(T)* (J $kmol^{-1}$ K^{-1}) is the heat capacity of the water, calculated according to Eq. S6, and T_o and T_f (298 and 343 K, respectively) are the initial and final temperatures during the heating process.

$$Cp(T) = a + b\,T + c\,T^2 + d\,T^3 + e\,T^4 \qquad \text{(S6)}$$

Where a (18.01528), b (276,370), c (- 2,090.1), d (8.125), and e (- 0.014116) are the parameters of the Eq. S6, which were retrieved from [22], and T (K) is the temperature.

Maintenance of the water temperature at 70 °C in the extraction vessel

The energy consumption for the maintenance of the water temperature at 70 °C in the extraction vessel per extraction cycle was calculated considering Eq. S7.

$$Q_{loss} = n_{stage}\, U\, A\, \Delta T\, t \quad \text{(S7)}$$

Where Q_{loss} (J cycle^{-1}) is the energy consumption for the maintenance of the water temperature in the extraction vessel per extraction cycle, U (0.55 W m^{-2} K^{-1}) is the overall heat transfer coefficient, A (m^2) is the surface area of the extraction vessel, ΔT (45 K) is the difference between the extraction temperature (343 K) and the room temperature (298 K), and t (s stage^{-1}) is the extraction time per stage.

The overall heat transfer coefficient used in this simulation was selected as a typical U value that assumes the use of heat exchange insulation materials in the equipment. These materials should be carefully selected in a subsequent stage of the scale-up Engineering project.

The surface area of the extraction vessel was estimated considering the height and diameter previously calculated. Such results are presented in Table S5.

Agitation of the liquid in the heating and mixing tanks

The energy required for the agitation of the heating and mixing tanks was estimated considering two dimensionless numbers: the impeller Reynolds number (Re_I) and the power number (N_P) [22], which were calculated according to Eqs. S8 and S9, respectively.

$$Re_I = \frac{(d^2\, N\, \rho_w)}{\mu_w} \quad \text{(S8)}$$

$$N_P = \frac{P}{\rho_w\, N^3\, d^5} \quad \text{(S9)}$$

Where d (m) is the impeller diameter, N (0.5 s^{-1}) is the rotational speed, ρ_w (987.7 kg m^{-3}) [22] is the average density of the water between 25 and 70 °C, μ_w (6.60 E-04 Pa s) [22] is the average viscosity of the water between 25 and 70 °C, and P (W) is the agitator power. The correlation of Re_I and N_P was obtained from a diagram from [22], and the impeller diameter was calculated considering Eq. S10, considering the suggestion by Hiraoka and collaborators [23].

$$\frac{d}{D} = 0.41 \quad \text{(S10)}$$

Where D (m) is the diameter of the tank.

Finally, the energy consumption per cycle was estimated considering the calculated agitator power and the time of agitation, which is presented in Table S7.

Table S7. Operational parameters considered for the estimation of the energy consumption for the agitation of the heating and mixing tanks.

Scenario	Conventional	Two-stage	Four-stage
**Heating tank**			
Number of stage (stage cycle^{-1})	1	2	4
Time of operation (min stage^{-1})	30	15	7.5

Mixing tank			
Number of stage (stage cycle^{-1})	-	1	1
Time of operation (min stage^{-1})	-	15	15

Agitation of the mixture in the extraction vessel

The energy required for the agitator of the extraction vessel was calculated similarly to the one calculated for the heating and mixing tanks, considering the dimensionless numbers of the impeller Reynolds (Re_I) and the power (N_P). Previously to the calculation, however, the agitation speed required to maintain the solid suspended in the extraction scenario was calculated, considering the settling velocity (v_s, terminal sedimentation velocity) of the yerba mate particles, calculated with the Eq. S11, considering a turbulent flow.

$$v_s = \sqrt{\frac{\left(4\, g\, d_p\, (\rho_s - \rho_w)\right)}{3\, \rho_w\, C_d}} \tag{S11}$$

Where g (9.81 m s^{-2}) is the gravity acceleration constant, d_p (3.45 x 10^{-4} m) is the diameter of the particle used in the extraction [21], and Cd (0.44) is the constant for spherical particles in turbulent flow.

The required agitation speed (N_{req}) was then calculated according to Eq. S12.

$$N_{req} \geq \frac{v_s}{\pi\, d} \tag{S12}$$

Once the required agitation speed is lower than the agitation speed of 0.5 s^{-1} considered for the heating and mixing tanks, the value of 0.5 s^{-1} was also considered in the power consumption estimation of the agitation on the extraction vessel.

Global economic analysis

Table S8. Equations for the estimation of the Total Capital Investment.

No	Parameter	Equation
**Direct costs**		
1	Equipment cost[1] (E)	-
2	Tool installation cost (T)	0.30 E
3	FOB Fee (F)	E + T
4	Insurance fee (IF)	0.01 F
5	Facility service fee (FS)	0.40 E
6	Total direct costs (D)	F + IF + FS
**Indirect costs**		
1	Engineering (Eng)	0.32 E
2	Contingency (Cont)	0.10 (D+Eng)
3	Total indirect costs (I)	Eng + Cont
**Fixed capital investment (FCI)**		**D + I**

**Working capital investment (WCI)**	**0.15 TCI**
**Total capital investment (TCI)**	**FCI + WCI**

Equipment costs assumed as the value estimated previously ([1]).

Table S9. Equations for the estimation of the Total Production Costs.

No	Parameter	Equation
**General expenses**		
1	Administration cost (Admin)	0.03 TCI
2	Distribution and marketing cost (DM)	0.05 TCI
3	Other expenses (O1)	0.05 TCI
4	Total general expenses (GE)	Admin + DM + O1
**Direct manufacturing cost**		
1	Raw material[1] (RM)	-
2	Labor[2] (L)	
3	Maintenance (Maint)	0.02 FCI
4	Electricity[3] (Elec)	
5	Total direct manufacturing cost (DMC)	RM + L + Maint + Elec
**Indirect manufacturing cost**		
1	Payroll overhead (PaO)	0.15 L
2	Plant overhead (PlO)	0.50 L
3	Total indirect manufacturing cost (IMC)	PaO + PlO
**Fixed manufacturing cost**		
1	Depreciation (Dep)	0.10 FCI
2	Taxes (Tax)	0.01 FCI
3	Packaging (Pack)	0.04 RM
4	Total fixed manufacturing cost (FMC)	Dep + Tax + Pack
**Manufacturing cost (MC)**		**DMC + IMC + FMC**
**Total production cost (TPC)**		**GE + MC**

Raw material (RM) calculated as the sum of the annual costs of yerba mate and water ([1]); Labor (L) calculated as the product of the number of employees (3 plant workers and one engineer) and the Brazilian average wage of 3,769 and 26,000 US$ year^{-1} for the plant workers and engineer, respectively ([2]); and Electricity (Elec) calculated as the product of the energy consumption of the main equipment previously calculated and the average energy cost in Brazil ([3]).

RESULTS

Preliminary extraction analysis

Table S10. Results on the concentration (C) and relative extraction efficiency (REE) of phenolic compounds from yerba mate on the preliminary extraction analysis.

Run	T (°C)	SLR (g 100 mL^{-1})	C[1] (mg GAE L^{-1})	REE[2] (mg GAE g^{-1})
1	50	0.5	446.8 ± 21.6	89.4 ± 4.4
2	50	1.5	1,224.0 ± 46.8	81.5 ± 2.9
3	60	1.0	891.7 ± 1.3	89.0 ± 1.5

4	70	0.5	493.2 ± 22.5	98.0 ± 4.7
5	70	1.5	1,345.1 ± 86.6	89.6 ± 5.7

Results expressed as Mean ± Standard Deviation. Concentration of the extract (1); and relative extraction efficiency (2).

Kinetics of the single-stage batch extraction

Table S11. Fitted parameters on the kinetics obtained at the best extraction conditions (70 °C and 0.5 g 100 mL^{-1}).

Parameter	Result
k_3 (min L mg^{-1} GAE)	1.43E-02
k_4 (L mg^{-1} GAE)	1.84E-03
MRE	0.025

Multi-stage batch extraction

Table S12. Relative Extraction Efficiency of phenolic compounds in the extract of yerba mate obtained in each stage of extraction for the different extraction scenarios.

**Scenario**	**Conventional**	**Two-stage**	**Four-stage**
Mass of yerba mate used in the extraction (g)	0.5	1.0	2.0
Number of stages	1	2	4
Total volume of water used in the extraction (mL)	100	200	400
Volume of water used in the individual stages (L)	0.1	0.1	0.1
**Concentration (mg GAE L^{-1})**			
1st stage	503.8 ± 7.4	976.6 ± 16.0	1,715.3 ± 9.0
2nd stage	-	76.3 ± 6.1	221.2 ± 2.0
3rd stage	-	-	28.3 ± 1.8
4th stage	-	-	7.4 ± 0.4
**Mass of extracted phenolic compounds (mg GAE)**			
1st stage	50.4 ± 0.7	97.7 ± 1.6	171.5 ± 0.9
2nd stage	-	7.6 ± 0.6	22.1 ± 0.2
3rd stage	-	-	2.8 ± 0.2
4th stage	-	-	0.7 ± 0.0
**Relative extraction efficiency (mg GAE g^{-1} yerba mate)**			
1st stage	100.8 ± 1.5	97.7 ± 1.6	85.8 ± 0.5
2nd stage	-	7.6 ± 0.6	11.1 ± 0.1
3rd stage	-	-	1.4 ± 0.1
4th stage	-	-	0.4 ± 0.0
**Global REE**	**100.8 ± 1.5**	**105.3 ± 2.2**	**98.6 ± 0.7**

Results expressed as Mean ± Standard Deviation.

Techno-economic and environmental analysis

Table S13. Equipment cost for the different extraction scenarios.

Scenario	Conventional	Two-stage	Four-stage
Heating tank	3,243	2,140	1,412
Extraction vessel	3,250	2,148	1,423
Mixing tank	-	1,642	1,642
**Total equipment cost (US$)**	**6,493**	**5,929**	**4,476**

Table S14. Raw material cost estimation.

Parameter	Result
Yerba mate processed per day (kg day^{-1})	25
Yerba mate processed per year[1] (kg $year^{-1}$)	6,250
Water used per day (m^3 day^{-1})	5.0
Water used per year[1] (m^3 $year^{-1}$)	1,250
Yerba mate cost[2] (US$ $year^{-1}$)	34,313
Water cost[3] (US$ $year^{-1}$)	6,085
**Raw material total cost (US$ $year^{-1}$)**	**40,398**

Consumption of the raw material per year was calculated considering an operation of 250 days $year^{-1}$ ([1]); yerba mate cost was calculated considering an average price of 5.49 US$ kg^{-1} yerba mate ([2]); and water cost was calculated considering an average price of 4.87 US$ m^{-3} water ([3]).

Table S15. Labor cost estimation.

Parameter	Result
Number of plant workers	3
Wage of the plant workers[1] (US$ $year^{-1}$ $worker^{-1}$)	3,769
Number of engineer	1
Wage of the engineer[2] (US$ $year^{-1}$ $worker^{-1}$)	26,000
**Labor total cost (US$ $year^{-1}$)**	**37,307**

Wage of the plant workers considered equal to Brazilian minimum wage in 2025, multiplied for 13 times ([1]); and wage of the engineer considered equal to the Brazilian minimum engineer wage in 2025, multiplied for 13 times ([2]).

Table S16. Energy consumption of specific steps of the extraction process for the extraction of phenolic compounds from yerba mate at different extraction scenarios.

Scenario	Conventional	Two-stage	Four-stage
Agitation of water in the heating tank (J $cycle^{-1}$)	5.78E+04	1.82E+04	5.74E+03
Heating of the water from 25 to 70 °C (J $cycle^{-1}$)	5.02E+10	5.02E+10	5.02E+10

Maintenance of the water temperature (70 °C) in the extraction vessel (J cycle^{-1})	1.88E+06	1.19E+06	7.52E+05
Agitation of the mixture in the extraction vessel (J cycle^{-1})	2.33E+05	7.37E+04	2.35E+04
Agitation of the extracts in the mixing tank (J cycle^{-1})	-	2.89E+04	2.89E+04
**Total energy consumption of the considered steps (J cycle^{-1})**	**5.02E+10**	**5.02E+10**	**5.02E+10**
**Total energy consumption excluding the water heating (J cycle^{-1})**	**2.17E+06**	**1.31E+06**	**8.10E+05**
**Energy saving[1] (J cycle^{-1})**	**-**	**8.63E+05**	**1.36E+06**
**Total energy consumption per year[2] (J year^{-1})**	**2.51E+13**	**2.51E+13**	**2.51E+13**
**Energy total cost[3] (US$ year^{-1})**	**21,909**	**21,909**	**21,908**

Energy saving calculated as the difference between the energy consumption of the evaluated scenario and the energy consumption of the conventional scenario ([1]); total energy consumption per year calculated considering an operation of 250 days year^{-1} ([2]); and energy total cost calculated considering an average price of 3.14E-03 US$ kWh^{-1} ([3]).

Table S17. Results on the estimation of the Total Capital Investment.

No	Parameter	Scenario		
		**Conventional**	**Two-stage**	**Four-stage**
**Direct costs**				
1	Equipment cost	6,493	5,929	4,476
2	Tool installation cost	1,948	1,779	1,343
3	FOB Fee	8,440	7,708	5,819
4	Insurance fee	84	77	58
5	Facility service fee	2,597	2,372	1,790
6	Total direct costs	11,122	10,157	7,668
**Indirect costs**				
1	Engineering	2,078	1,897	1,432
2	Contingency	1,320	1,205	910
3	Total indirect costs	3,398	3,103	2,342
**Fixed capital investment (FCI)**		**14,519**	**13,260**	**10,010**
**Working capital investment (WCI)**		**2,562**	**2,340**	**1,766**
**Total capital investment (TCI)**		**17,082**	**15,600**	**11,777**

Table S18. Results on the estimation of the Total Production Costs.

No	Parameter	Scenario		
		**Conventional**	**Two-stage**	**Four-stage**
**General expenses**				
1	Administration cost	512	468	353

2	Distribution and marketing cost	854	780	589
3	Other expenses	854	780	589
4	Total general expenses	2,221	2,028	1,531
**Direct manufacturing cost**				
1	Raw material	40,398	40,398	40,398
2	Labor	37,307	37,307	37,307
3	Maintenance	290	265	200
4	Electricity	21,909	21,909	21,908
5	Total direct manufacturing cost	99,904	99,879	99,813
**Indirect manufacturing cost**				
1	Payroll overhead	5,596	5,596	5,596
2	Plant overhead	18,654	18,654	18,654
3	Total indirect manufacturing cost	24,250	24,250	24,250
**Fixed manufacturing cost**				
1	Depreciation	1,452	1,326	1,001
2	Taxes	145	133	100
3	Packaging	1,616	1,616	1,616
4	Total fixed manufacturing cost	3,213	3,075	2,717
**Manufacturing cost (MC)**		**127,367**	**127,203**	**126,780**
**Total production cost (TPC)**		**129,587**	**129,231**	**128,311**

REFERENCES

[19] Manufacturers, Suppliers, Exporters & Importers from the world's largest online B2B Marketplace. https://www.alibaba.com/, 2025 (accessed June 21, 2025).

[21] Domingues, L.H.P.; Arantes, M.S.T.; Marques, G.S.; Haminiuk, C.W.I.; Fontana, E.; da Silva, V.R. Extraction of bioactive compounds from yerba mate (*Ilex paraguariensis St.-Hil.*) leaves by packed-bed extractor using hot water as solvents: Kinetics study and mathematical modeling. AIMS Mol. Sci. **2024**, 11, 42–60. https://doi.org/10.3934/molsci.2024003.

[22] Green, D.W.; Southard, M. Perry's Chemical Engineering Handbook, ninth ed., McGraw Hill, **2019**.

[23] Hiraoka, S.; Kato, Y.; Tada, Y.; Ozaki, N.; Murakami, Y.; Lee, Y.S. Power Consumption and Mixing Time in an Agitated Vessel with Double Impeller. Chem. Eng. Res. Des. **2001**, 79, 805–810. https://doi.org/10.1205/02638760152721613.
